# Integrative cross-species analysis reveals conserved and unique signatures in fatty skeletal muscles

**DOI:** 10.1038/s41597-024-03114-5

**Published:** 2024-03-12

**Authors:** Liyi Wang, Yanbing Zhou, Yizhen Wang, Tizhong Shan

**Affiliations:** 1 College of Animal Sciences, Zhejiang University, Hangzhou, China; 2 Key Laboratory of Molecular Animal Nutrition (Zhejiang University), Ministry of Education, Hangzhou, China; 3 Key Laboratory of Animal Feed and Nutrition of Zhejiang Province, Hangzhou, China

**Keywords:** Mechanisms of disease, Ageing, Transcriptomics

## Abstract

Fat infiltration in skeletal muscle is now recognized as a standard feature of aging and is directly related to the decline in muscle function. However, there is still a limited systematic integration and exploration of the mechanisms underlying the occurrence of myosteatosis in aging across species. Here, we re-analyzed bulk RNA-seq datasets to investigate the association between fat infiltration in skeletal muscle and aging. Our integrated analysis of single-nucleus transcriptomics in aged humans and Laiwu pigs with high intramuscular fat content, identified species-preference subclusters and revealed core gene programs associated with myosteatosis. Furthermore, we found that fibro/adipogenic progenitors (FAPs) had potential capacity of differentiating into PDE4D^+^/PDE7B^+^ preadipocytes across species. Additionally, cell-cell communication analysis revealed that FAPs may be associated with other adipogenic potential clusters via the COL4A2 and COL6A3 pathways. Our study elucidates the correlation mechanism between aging and fat infiltration in skeletal muscle, and these consensus signatures in both humans and pigs may contribute to increasing reproducibility and reliability in future studies involving in the field of muscle research.

## Introduction

Skeletal muscle is one of the largest organs in the human body, accounting for approximately 40% of total body mass and playing an important role in locomotion, glucose and lipid homeostasis. Myogenesis in skeletal muscle involves several biological processes including activation of resident myogenic precursor satellite cells (SCs), proliferation and differentiation of the myoblast, fusion of myocytes, and finally maturation of myofibers^1^. This process is regulated by many myogenic transcription factors such as paired box 7 (Pax7), myogenic differentiation (MyoD), myogenic factor 5 (Myf5), and myogenin (MyoG)^2^. The homeostatic maintenance of skeletal muscle is also regulated by the niche compartment, comprising various cell types, such as SCs, endothelial cells (ECs), smooth muscle cells, fibro/adipogenic progenitors (FAPs), tenocytes, and immune cells^3–5^. Fat infiltration in skeletal muscle (intramuscular fat (IMF) deposition, also known as myosteatosis) is the pathologic fat accumulation in skeletal muscle with poor metabolic and musculoskeletal health, which is now considered a distinct disease from sarcopenia^6,7^. However, in animal production, IMF deposition is positively associated with meat quality, including meat color, tenderness, and juiciness. The increasing risk of muscular dysfunction and metabolic disorders consistently accompanies myosteatosis, encompassing conditions such as aging, sarcopenia, diabetes, and obesity^8,9^. Hence, an in-depth understanding of the occurrence mechanism underlying fat infiltration in skeletal muscle is of scientific and clinical importance.

Fat infiltration in skeletal muscle is regulated by many triggering factors, including aging, diseases, muscle injury, disuse and inactivity, which is modulated by many regulators, genes and signaling pathways^10,11^. Age-related decline, also known as “sarcopenia”, entails reduced mass and strength of skeletal muscle, significantly contributing to age-related metabolic dysfunction, comorbidities, and premature death^12^. Myosteatosis is also recognized as a standard feature of aging that is associated with diminished muscle strength, altered muscle architecture, impaired muscle contraction, and reduced muscle capacity^13,14^. It has been observed that fat infiltration occurs in the skeletal muscles of elderly individuals and aged animals^15–17^. However, the functional changes of multinucleated muscle cells during skeletal muscle aging and the transcriptomic programs of fat infiltration in skeletal muscle remain largely unknown.

In skeletal muscle, mesenchymal stem cells (MSCs) play a role in IMF formation and PDGFRα^+^ mesenchymal progenitors, distinct from SCs, have been identified as capable of forming ectopic fat cells^18^. FAPs, which mainly express platelet-derived growth factor receptor α (PDGFRα)^+^ cells, can proliferate and differentiate into adipose and/or fibrous tissue after damage in skeletal muscle^19,20^. Historically, FAPs are the main source of IMF^21,22^ and recent studies have found that MME^+^ FAPs are highly adipogenic and are reduced in fatty infiltrated human muscle^23^. However, there are other skeletal muscle resident cells that contribute to IMF formation in pathological conditions. Fibroblasts, side population cells (SPs, also called myoendothelial cells), pericytes and mesoangioblasts, and PW1^+^/Pax7^−^ interstitial cells (PICs) also contribute to the fat deposition and accumulation in the damaged and inflamed skeletal muscle^24^. Moreover, several factors have been reported to activate the transdifferentiation potential of SCs into adipocytes in skeletal muscle, such as genes, nutrients, age, regeneration and oxidative stress^25^. Specially, a recent study found that a subpopulation of myeloid-derived cells may induce fat infiltration in skeletal muscle^26^. With the rapid development of high-throughput sequencing technology, single-nucleus RNA-seq (snRNA-seq) can now be used for profiling the transcriptomes of mature muscle cells such as myotubes or myofiber at the whole cell or nucleus level. Recently, Perez *et al*. found many cell types including MSCs, FAPs, and ECs were identified in *vastus lateralis* of old people by using snRNA-seq^27^. Similarly, in animals, Jing *et al*. identified adipocyte clusters and MSCs, FAPs, ECs, and pericytes in the aged muscle of monkeys^28^. In mice, adipocyte clusters can also be found in 24-month *tibialis anterior*^5^. The pig has become an increasingly utilized animal model for biomedical research involving humans and human diseases due to its physiological structure, which is similar to that of humans^29,30^. Laiwu pig is an excellent Chinese local pig species known for its high IMF content. Our previous study has demonstrated the heterogeneity of fat deposition in multinucleated skeletal myofibers in pigs^31^. Hence, Laiwu pigs can be used as an alternative animal model to study the transcriptomic dynamics of the association between fat deposition in muscle and aging. However, the differences between aged and high IMF content muscle have rarely been investigated.

In this study, we performed a comprehensive integrated analysis of publicly available expression profiles of aged human and Laiwu pigs with high IMF content. Our investigation aimed to elucidate the relationship between myosteatosis and aging using public RNA-seq datasets. We compared the myonuclear heterogeneity and transcriptomic profiles in skeletal muscle across species and identified core gene programs. Moreover, we unveiled conserved and differential genes associated with myosteatosis between different species. Our findings will provide novel insights into understanding the cytological and molecular mechanism of fat infiltration in skeletal muscle. This understanding may prove crucial for the treatment of muscle-related diseases and for bolstering the utilization of pigs as animal models in biomedical research, particularly in studies of human diseases.

## Results

### Aging is accompanied by fat infiltration in skeletal muscle

**Fig. 1:**
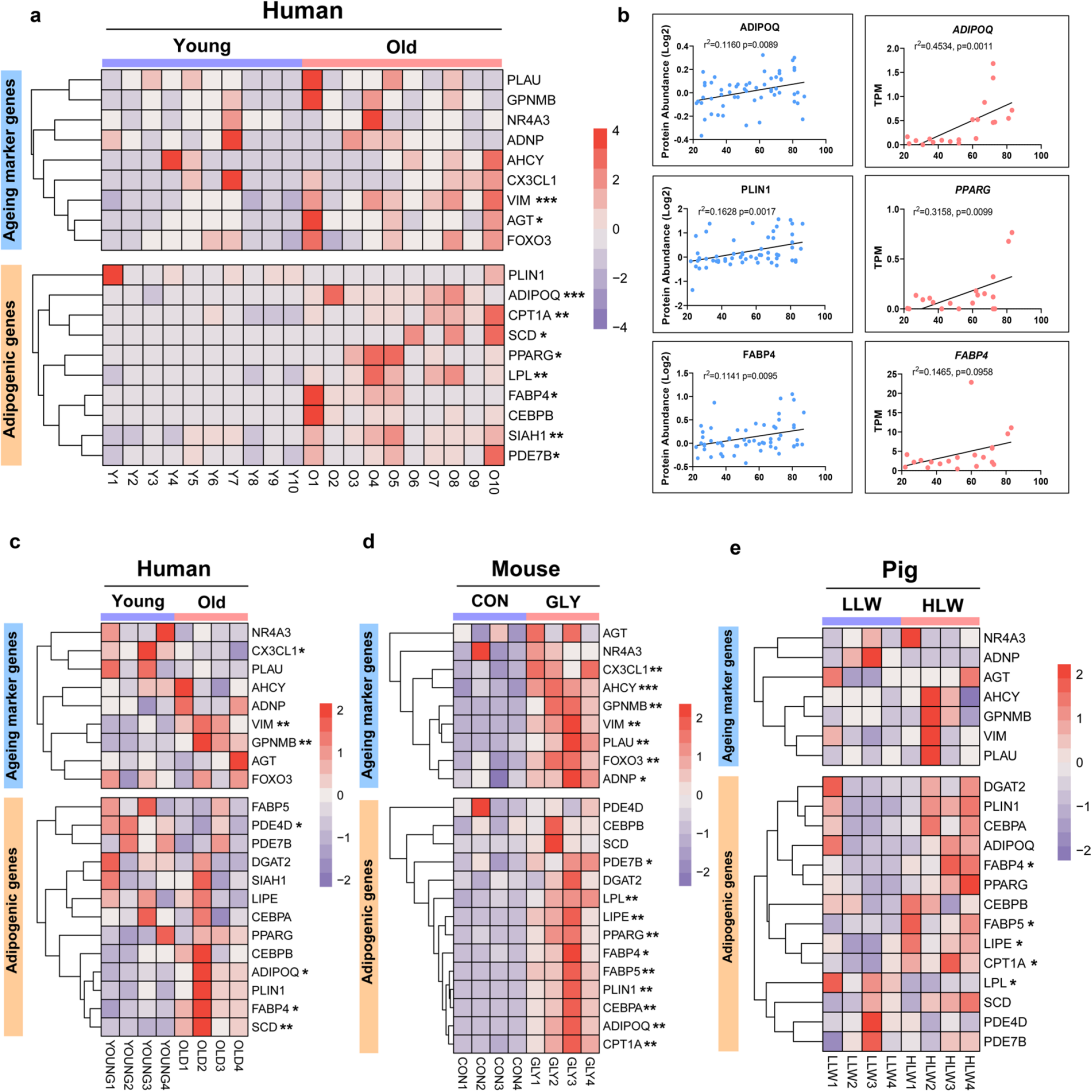
The association between fat infiltration and aging across different species. (**a**) Heatmap of select aging marker genes and adipogenic genes in young and aged human muscles. *P < 0.05, **P < 0.01, ***P < 0.001, two-tailed Student’s t-test. n = 10 for each group. (**b**) Log2 protein abundance of select adipogenic proteins (ADPIOQ, PLIN1, and FABP4) and TPM value of select adipogenic genes (*ADPIOQ*, *PPARG*, and *FABP4*) in young and aged human muscles. Simple linear regression was shown for age (x axis) and protein or genes (y axis) correlation, unadjusted p-values by two-tailed test and r squared were shown. n = 58 for protein abundance and n = 20 for mRNA expression. (**c**) Heatmap of select aging marker genes and adipogenic genes in young and aged human muscles. *P < 0.05, **P < 0.01, ***P < 0.001, two-tailed Student’s t-test. n = 4 for each group. (**d**) Heatmap of select aging marker genes and adipogenic genes in control and GLY-injected mouse muscles. *P < 0.05, **P < 0.01, ***P < 0.001, two-tailed Student’s t-test. n = 4 for each group. (**e**) Heatmap of select aging marker genes and adipogenic genes in low IMF content and high IMF content pig muscles. *P < 0.05, **P < 0.01, ***P < 0.001, two-tailed Student’s t-test. n = 4 for each group. Reprinted from^34^ Copyright 2024, Elsevier; under the Creative Commons CC BY License.

To explore the associations between aging and fat infiltration in skeletal muscle across species, we first re-analyzed published RNA-seq and proteomics datasets about aged human muscle^32^. These results showed the aging marker genes (*VIM* and *AGT* as reported previously^33^) were significantly increased in old human muscle, while adipogenic genes (*ADIPOQ, FABP4, PPARG, CPT1A* and *SCD*) also significantly upregulated (Fig. 1a). Correlation analysis identified that the protein expression of ADIPOQ, PLIN1, and FABP4 and the gene expression of *ADIPOQ*, *PPARG*, and *FABP4* were positively related to age^34^ (Fig. 1b). Similarly, another RNA-seq datasets focusing on aged male muscles also showed the significant regulation of aging marker genes (*VIM*, *CX3CL1*, and *GPNMB*) and adipogenic genes (*ADIPOQ, FABP4*, and *SCD*) (Fig. 1c). In GLY-injected mouse model that recapitulates fat infiltration in skeletal muscle^26^, the increased expression of adipogenic genes (*ADIPOQ, FABP4, PPARG, CPT1A* and *SCD*) consistently accompanied the elevation of aging marker genes (*VIM*, *CX3CL1*, *GPNMB*, *ACHY*, *PLAU*, *FOXO3*, and *ADNY*) (Fig. 1d). Interestingly, based on previous study^31^, we also observed an increased tendency in the expression of aging marker genes in high IMF content pigs (Fig. 1e). These results indicate greater similarities in associations between aging and fat infiltration in skeletal muscle among larger mammals.

### snRNA-seq identified distinct cell populations in aged human and high IMF content pig muscles

**Fig. 2:**
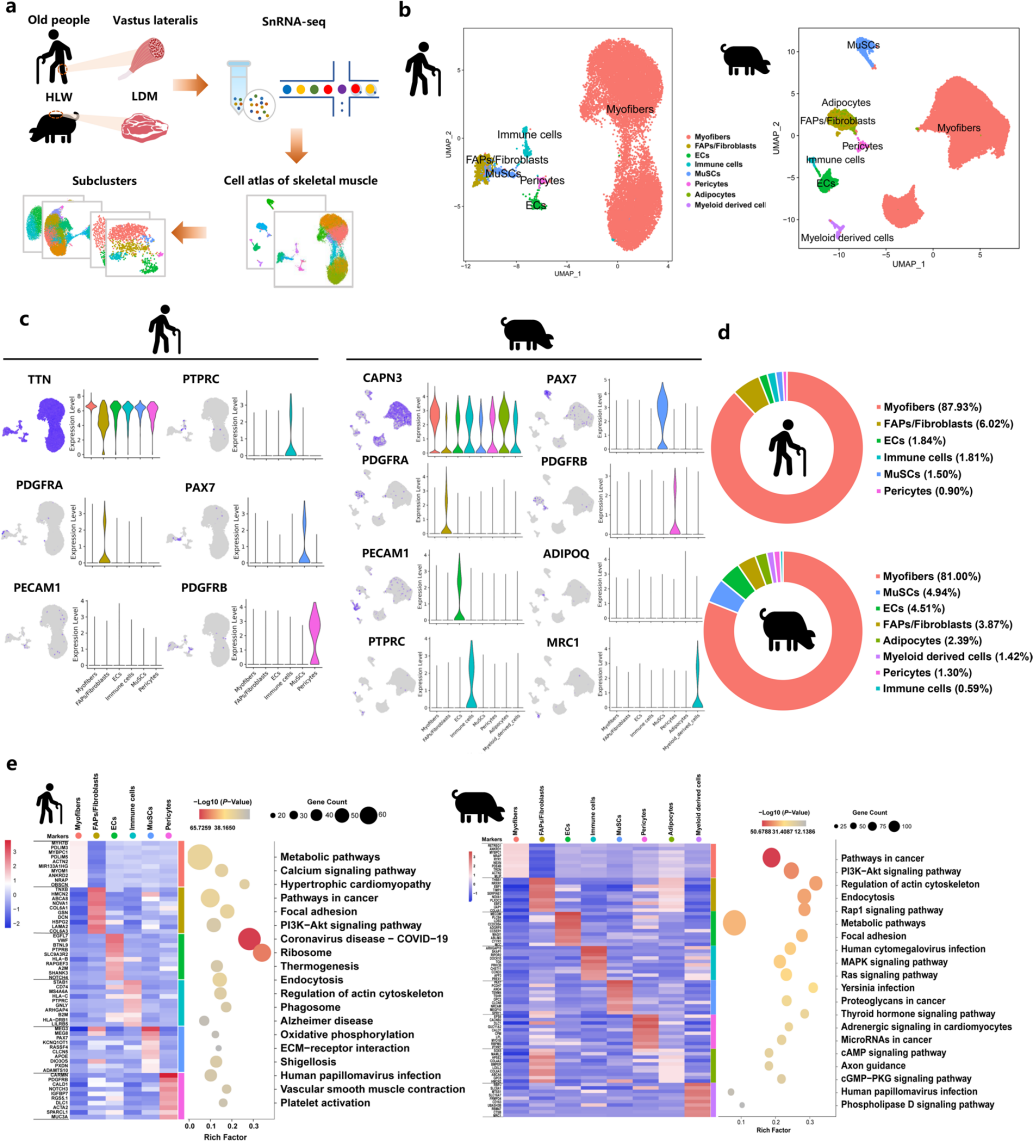
SnRNA-seq identifies distinct cell populations of aged human and high IMF content pig muscles. (**a**) Scheme of the experimental design for snRNA-seq on different muscle nuclei. (**b**) UMAP visualization of all of the isolated single nuclei from aged human and high IMF content pig muscles colored by cluster identity. (**c**) UMAP and violin plot displaying the expression of selected marker genes for each cluster in different species. (**d**) Nuclear proportion in each cluster in aged human and high IMF content pig muscles. Each cluster is color-coded. (**e**) Left, heatmap showing the top 10 most DEGs by bimod difference analysis algorithm (*P*-Value < 0.01, Log2FC ≥ 0.26) between cell types identified. Right, KEGG enrichment for marker genes of each cell type in different species. Each lane represents a subcluster.

To investigate the cell dynamics of aged muscle and further explore the specific association between aging and fat infiltration in skeletal muscle at the cytological level, we re-analyzed published snRNA-seq datasets about aged human and high IMF content pig muscles^27,31^ (Fig. 2a). Cell Ranger analyses showed the estimated number of cells, fraction of reads in cells, mean reads per cell, median genes per cell, and median UMI counts per cell in this study (Supplementary Fig. 1b). Using the Seurat package (v3.1.1), aggregated and normalized snRNA-seq data were then subjected to clustering to identify the cell types, as shown in Uniform Manifold Approximation and Projection (UMAP) plots (Fig. 2b). Based on the expression of lineage specific markers as previously reported^4,5^, we identified 6 different clusters of nuclei, including myofibers (*TTN*), FAPs/fibroblasts (*PDGFRA*), ECs (*PECAM1*), immune cells (*PTPRC*), muscle satellite cells (MuSCs) (*PAX7*), and pericytes (*PDGFRB*) in human muscles (Fig. 2c). In pigs, we found other 2 specific clusters of nuclei, including adipocytes (*ADIPOQ*) and myeloid derived cells (*MRC1*) (Fig. 2c). Next, we analyzed the difference in cell populations in different species. Compared with the aged human, the high IMF content pig muscles had two specific clusters: adipocytes nuclei (2.39%) and myeloid derived cells (1.42%) (Fig. 2d). The heatmap showed the top 10 most variably expressed genes and the top three KEGG enrichment for marker genes between the 9 cell clusters in different species (Fig. 2e). These results suggested that cell populations had significant differences between aged human and high IMF content pig muscles.

### Inter-species comparison identified core gene program in skeletal muscle

**Fig. 3:**
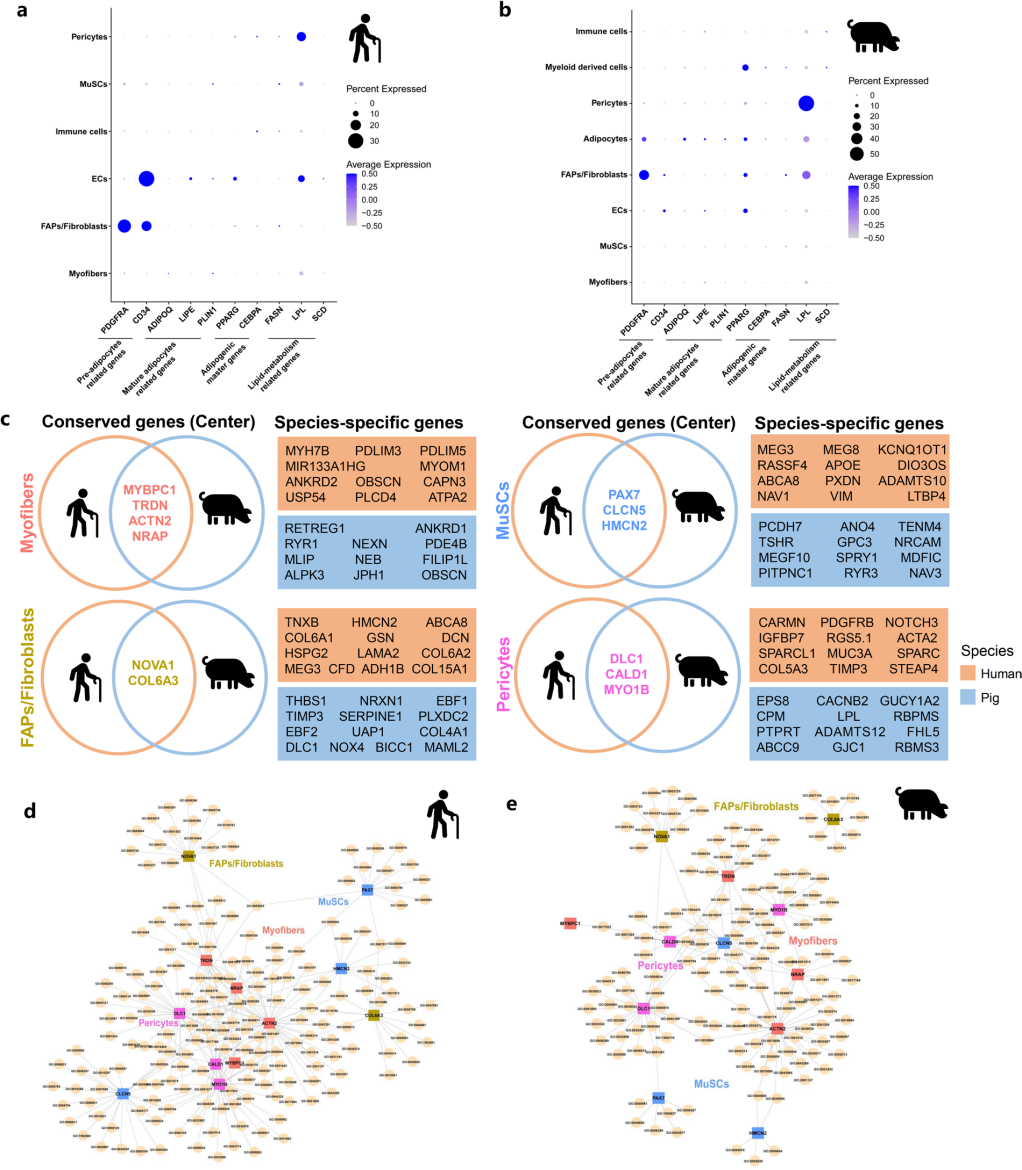
Inter-species comparison identifies core gene program of fat infiltration in skeletal muscle. (**a**) Dotplot showing the expression of preadipocyte-related genes, mature adipocyte marker genes, adipogenic master genes, and lipid metabolism-related genes in aged human muscles. (**b**) Dotplot showing the expression of preadipocyte-related genes, mature adipocyte marker genes, adipogenic master genes, and lipid metabolism-related genes in high IMF content pig muscles. (**c**) Conserved and species-specific genes of top 15 expressed genes in different cell clusters. The conserved genes among all species were marked in the left center of circles with different colors showing each cell types. Species-specific genes were located at the right rectangles with different colors showing each species. (**d**) The association between conserved genes in aged human muscles. (**e**) The association between conserved genes in high IMF content pig muscles.

To determine the difference in fat infiltration in skeletal muscle of different species, we examined the expression of adipogenesis- and lipid metabolism-related genes. As shown in Fig. 3a, pre-adipocytes related genes (*PDGFRA*^35^ and *CD34*^36^) were expressed in FAPs/Fibroblasts and ECs cell types. The expression of mature adipocyte marker genes (*ADIPOQ*, *PLIN1*, and *LIPE*) and adipogenic master genes (*PPARG*, and *CEBPA*) was enriched in ECs cell types, and lipid metabolism-related genes (*FASN*, *LPL*, and *SCD*) were expressed in ECs and pericytes cell types in aged human muscles. In pigs, we found that the expression of pre-adipocytes related genes (*PDGFRA* and *CD34*) was also enriched in FAPs/Fibroblasts and ECs cell types, while mature adipocyte marker genes (*ADIPOQ*, *PLIN1*, and *LIPE*) and adipogenic master genes (*PPARG*, and *CEBPA*) were expressed in adipocytes cell types, and lipid metabolism-related genes (*FASN*, *LPL*, and *SCD*) were expressed in ECs and myeloid derived cells (Fig. 3b). Additionally, we summarized the representative genes conserved in different species, with species-specificity for myofibers, FAPs/Fibroblasts, MuSCs, and pericytes in the top 15 expressed genes (Fig. 3c). There were 4, 2, 3 and 3 conserved genes existed in all species in myofibers (*MYBPC1*, *TRDN*, *ACTN2*, and *NRAP*), FAPs/Fibroblasts (*NOVA1* and *COL6A3*), MuSCs (*PAX7*, *CLCN5*, and *HMCN2*) and pericytes (*DLC1*, *CALD1*, and *MYO1B*), respectively (Fig. 3c). The network plot showed the interaction among these conserved genes in aged human and high IMF content pig muscles (Fig. 3d,e). Together, these data revealed the conserved cell populations and genes of fat infiltration in skeletal muscle across species.

### Clustering analysis identified subpopulations and transcriptional dynamics of myofibers

**Fig. 4:**
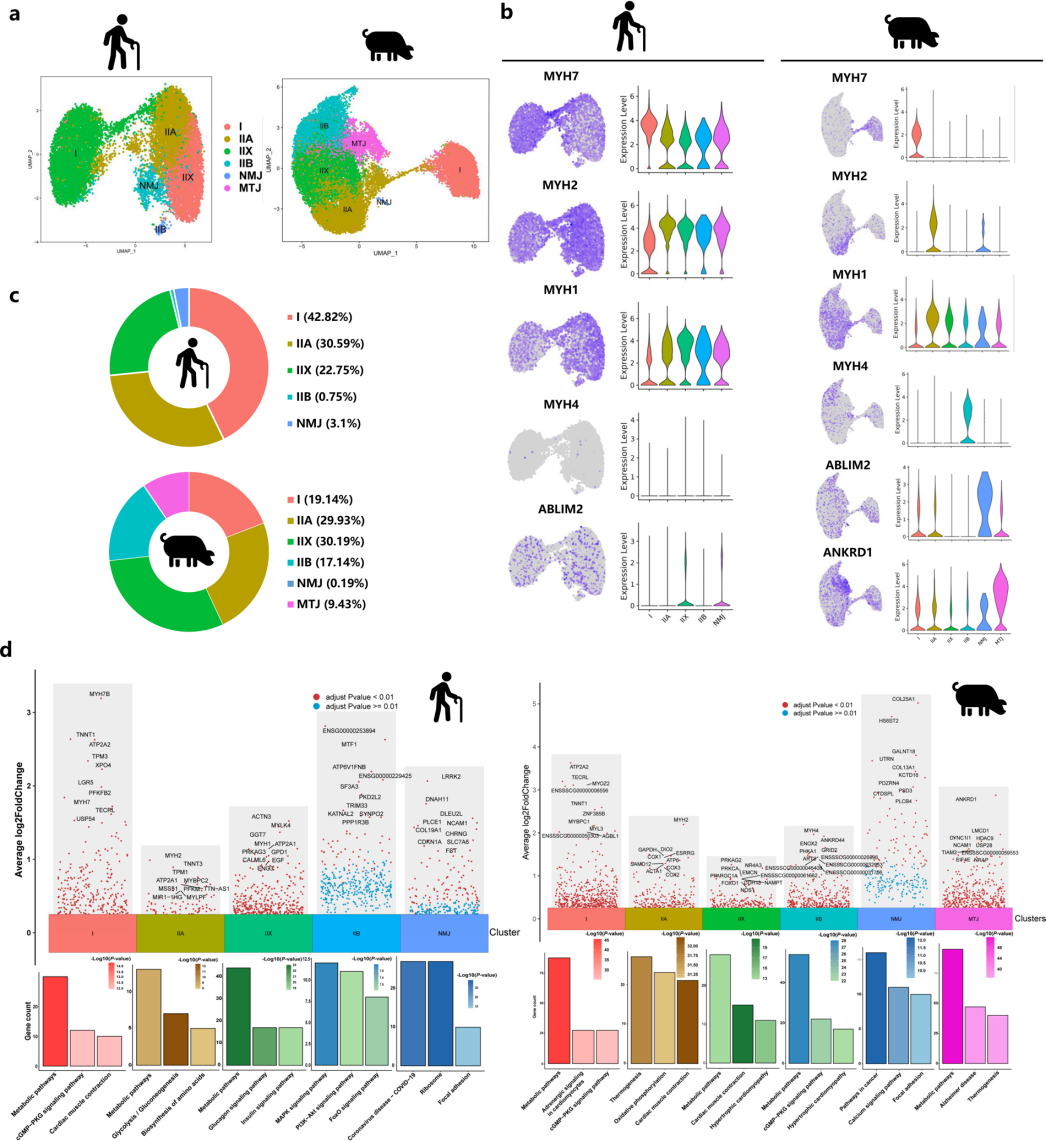
Clustering and transcriptional dynamics of myofibers nuclei in different species. (**a**) UMAP plot showing six subclusters of the isolated single nuclei from aged human and high IMF content pig muscles. (**b**) UMAP and violin plot displaying the expression of selected marker genes for each subcluster of myofibers nuclei. (**c**) Nuclei proportion in each subcluster in different species. Each cluster is color-coded. (**d**) Volcano plot representing the top 10 most DEGs by bimod difference analysis algorithm (*P*-Value < 0.01, Log2FC ≥ 0.26) between cell subclusters identified and KEGG enrichment for marker genes of each cell type in different species. I, type I myonuclei; IIA, type IIa myonuclei; IIX, type IIx myonuclei; Type IIB, type IIb myonuclei; MTJ, myotendinous junction nuclei; NMJ, neuromuscular junction nuclei.

To identify the heterogeneity of myofiber cell type, we performed a subcluster analysis and explored the heterogeneity of myofiber nuclei. UMAP plots showed the distribution in different subclusters of myofiber nuclei (Fig. 4a). We characterized 5 and 6 subclusters in myofiber nuclei of humans and pigs according to common signature genes combined with the distribution in UMAP plot, respectively, including type I myonuclei (*MYH7*), type IIa myonuclei (*MYH2*), type IIx myonuclei (*MYH1*), type IIb myonuclei (*MYH4*), neuromuscular junction (NMJ, *ABLIM2*)^37^ and myotendinous junctions (MTJ, *ANKRD1*)^5^ (Fig. 4b and Supplementary Fig. 2a,b). In the proportion of these subclusters, we found that in aged human muscles, the percentage of type I myonuclei was higher than high IMF content pig muscles (42.82% *vs*. 19.14%) while the percentage of type IIb myonuclei was lower (0.75% *vs*. 17.14%) (Fig. 4c). As shown in Fig. 4d, the volcano plot showed the top 10 most differently expressed genes (DEGs) between the cell subclusters. We found that *MYH7*, *MYH2*, and *MYH1* were significantly expressed in type I myonuclei, type IIa myonuclei, and type IIx myonuclei in aged human muscles, while *MYH2*, *MYH4*, and *ANKRD1* were significantly expressed in type IIa myonuclei, type IIX myonuclei, and MTJ myonuclei in high IMF content pig muscles (Fig. 4d). Besides, functional enrichment analyses using KEGG pathways revealed a significant enrichment of the signalling pathways in different species (Fig. 4d). These data demonstrated the significant heterogeneity in myofiber nuclei in aged human and high IMF content pig muscles.

### Characterization of FAPs across species through clustering and pseudotime analysis

**Fig. 5:**
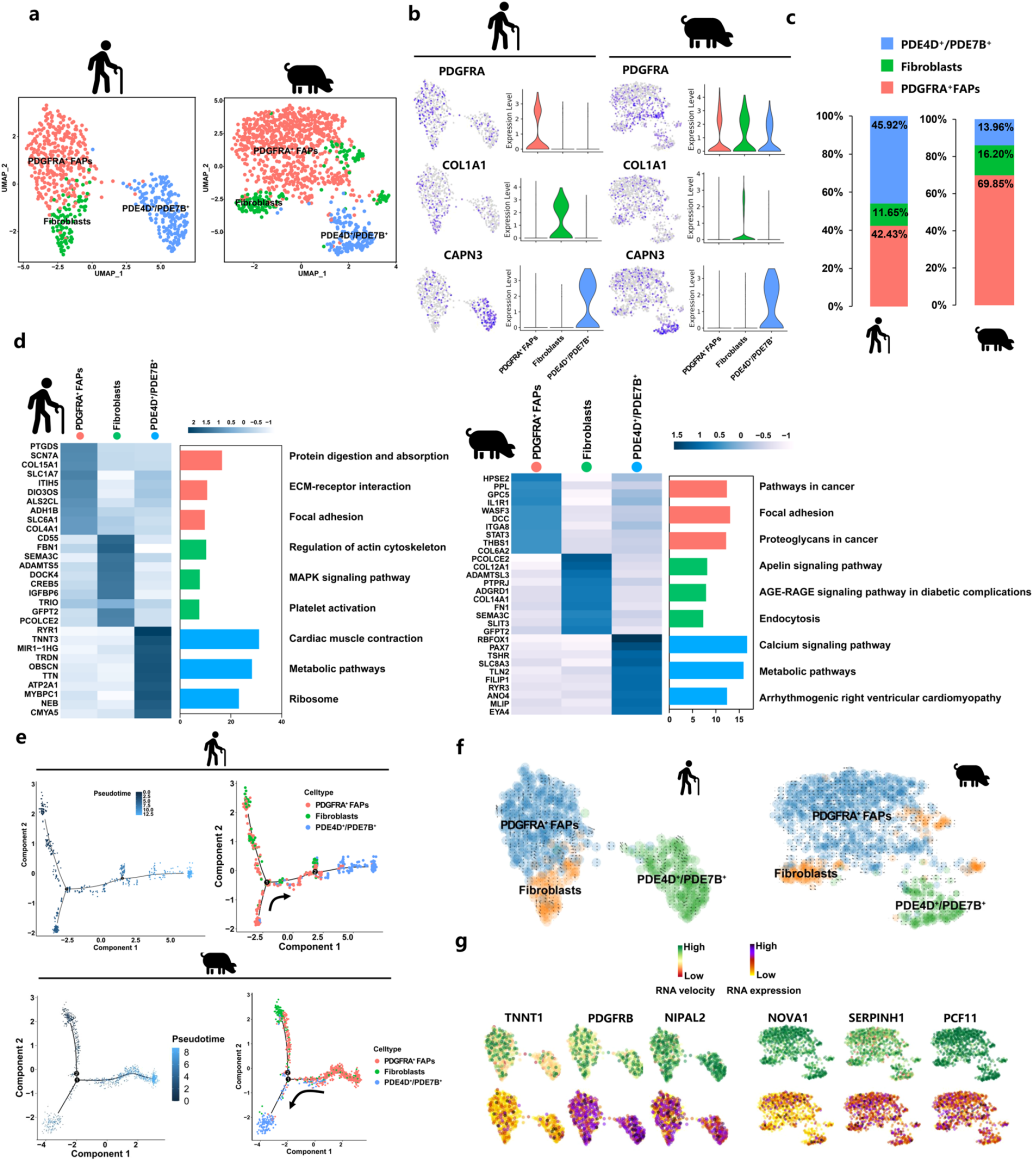
Clustering and pseudotemporal trajectories of FAPs nuclei in aged human and high IMF content pig muscles. (**a**) UMAP plot showing three subclusters of the isolated single nuclei from HLW and LLW muscle. (**b**) UMAP and violin plot displaying the expression of selected marker genes for each subcluster of nuclei. (**c**) Nuclear proportion in each subcluster in different species. Each cluster is color-coded. (**d**) Left, heatmap showing the top 10 most DEGs by bimod difference analysis algorithm (*P*-Value < 0.01, Log2FC ≥ 0.26) between cell types identified. Right, KEGG enrichment for marker genes of each cell type in different species. Each lane represents a subcluster. (**e**) Pseudotime ordering of all of the FAP/fibroblast nuclei of subcluster PDGFRA^+^ FAPs, fibroblasts, and PDE4D^+^/PDE7B^+^. The horizontal and vertical coordinates are two principal components respectively in two dimensions. Each dot represents one nucleus (color-coded by its identity), and each branch represents one cell state. Pseudotime is shown colored in a gradient from dark to light blue, and the start of pseudotime is indicated. Activation of the PDGFRA^+^ FAPs cluster can lead to PDE4D^+^/PDE7B^+^ fate. (**f**) Unsupervised pseudotime trajectory of the three subtypes of FAPs by RNA velocity analysis. Trajectory is colored by cell subtypes. The arrow indicates the direction of cell pseudo-temporal differentiation. (**g**) Distribution of marker genes of the three subtypes on the UMAPs based on RNA velocity analysis calculated differences by t-test.

To investigate the cellular origin of fat infiltration in skeletal muscle, we then analysed pre-adipocyte nuclei. Our previous studies have shown that FAPs have the capacity to differentiate into IMF cells^31^. Therefore, we applied subcluster and pseudotime analysis on FAPs/fibroblasts. Similar to our previous study, we also identified 3 subclusters in FAPs/fibroblasts of aged human and high IMF content pig muscles (Fig. 5a) according to the expression of specific markers, including PDGFRA^+^ FAPs (*PDGFRA*), fibroblasts (*COL1A1*), and PDE4D^+^/PDE7B^+^ subclusters (*NEB* and *PDE4D*) (Fig. 5b and Supplementary Fig. 3a,b). In aged human muscles, we found the proportion of nuclei in PDE4D^+^/PDE7B^+^ (45.92% *vs*. 13.96%) was higher than high IMF content pig muscles and the proportion of nuclei in PDGFRA^+^ FAPs (42.43% *vs*. 69.85%) was lower (Fig. 5c). As shown in Fig. 5d, the heatmap expressed the top 10 DEGs between the three cell subclusters and KEGG enrichment analyses revealed a significant enrichment of the signalling pathways in human and pigs. Interestingly, we found that focal adhesion was enriched in PDGFRA^+^ FAPs subclusters and the metabolic pathway was enriched in PDE4D^+^/PDE7B^+^ subclusters in aged human and high IMF content pig muscles (Fig. 5d). To further explore the differentiated trajectory of FAPs, we performed a trajectory analysis and RNA velocity analysis of FAPs/fibroblasts using Monocle 3 and scVelo (Supplementary Fig. 3c). As shown in Fig. 5e,f, PDGFRA^+^ FAPs could differentiate into PDE4D^+^/PDE7B^+^ subclusters in both aged human and high IMF content pig muscles. The pseudotemporal heatmap showed gene expression dynamics at Point 2 (Supplementary Fig. 3d). These data indicated that aged muscle and high IMF content muscle had some similar characteristics and FAPs might have the capacity of differentiating to PDE4D^+^/PDE7B^+^ preadipocytes in different species (Fig. 5g and Supplementary Fig. 3e,f).

### Cell-cell communication analysis revealed the networks of cell types in muscles of different species

**Fig. 6:**
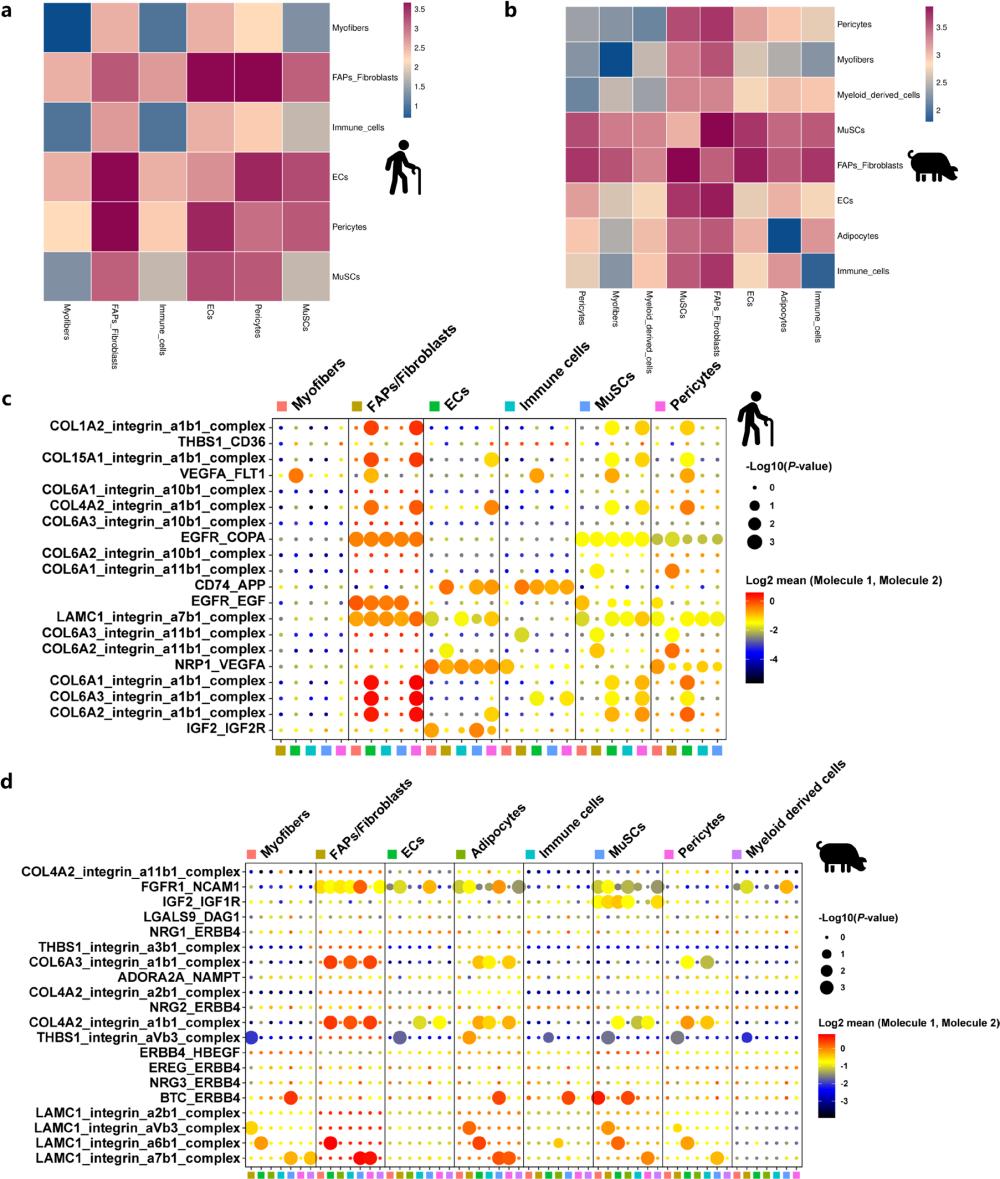
Dynamics of cell-cell communication networks. (**a**) Heatmap showing the number of protein interactions between pairs of different cell types (*P*-Value ≤ 0.05) counted by CellPhoneDB in aged human muscles. (**b**) Heatmap showing the number of protein interactions between pairs of different cell types (*P*-Value ≤ 0.05) counted by CellPhoneDB in high IMF content pig muscles. (**c**) Dotplot representing the gene expression and significance of the receptor-ligand relationship in different cell populations in aged human muscles. The top 20 receptor pairs after the sum of mean values and the top 30 cell pairs drawn. The depth of the color in the picture represents the strength of the corresponding interaction. Dot size indicates *P*-value generated by permutation test, colored by mean (mean = (ligand gene expression in ligand cells + receptor gene expression in recipient cells)/2). The larger the circle, the smaller the *P* value of the relationship in the corresponding cell population, the more significant it is. (**d**) Dotplot representing the gene expression and significance of the receptor-ligand relationship in different cell populations in high IMF content pig muscles. The top 20 receptor pairs after the sum of mean values and the top 30 cell pairs drawn. The depth of the color in the picture represents the strength of the corresponding interaction. Dot size indicates *P*-value generated by permutation test, colored by mean (mean = (ligand gene expression in ligand cells + receptor gene expression in recipient cells)/2). The larger the circle, the smaller the *P* value of the relationship in the corresponding cell population, the more significant it is.

To further explore the networks of cell types in fatty filtration muscles, we performed cell-cell communication analysis on 6 and 8 clusters in muscle nuclei, respectively. Interestingly, as shown in Fig. 6a, FAPs/fibroblasts predominantly interacted with ECs and pericytes in aged human muscles. In high IMF content pig muscles, FAPs/fibroblasts predominantly interacted with FAPs/fibroblasts, MuSCs, and ECs (Fig. 6b). Dotplot represented more robust communication from FAPs/fibroblasts to other cell types through EGF, COL4A2, and COL6A3 pathways in aged human muscles (Fig. 6c). In high IMF content pig muscles, we found more robust communication from adipocytes and FAPs/fibroblasts to other cell types through COL6A3, COL4A2, and FGF pathways (Fig. 6d). These data indicated that those cell types with adipogenic potential such as FAPs/fibroblasts, ECs, and pericytes interact with each other and these tight association may through COL4A2 and COL6A3 pathways across species. However, the detailed regulation of these cell populations and species-specific profiling on fat infiltration in skeletal muscle needs to be further explored.

## Discussion

Aging is associated with physiological, metabolic, and functional dysfunction, partly due to age-related changes in body composition. In particular, aging is accompanied by fat infiltration in skeletal muscle, which may serve as a significant contributor to muscular dysfunction.

Previous studies have indicated that fat infiltration and IMF deposition often occur in the skeletal muscles of elderly individuals as well as aged animals. In older African American women, a prior study revealed an age-related remodeling of body composition with a reduction in skeletal muscle and an increase intramuscular adipose tissue (IMAT) content, which suggested that aging-induced fat infiltration in skeletal muscle is always correlated with sarcopenia^8^. In our study, we discovered the levels of adipogenesis-related genes and protein (ADIPOQ, FABP4, and PLIN1) were positively with age based on the bulk RNA-seq and quantitative proteomic analysis in skeletal muscle of human^32,38,39^. Similarly, aging is also related to IMAT and IMF deposition in skeletal muscle of animal models. In aged male Sprague-Dawley rats, high-fat diet-induced muscle loss is associated with greater deposition of long-chain fatty acid esters^16^. For growing pigs, IMF content also increases linearly with age^17^. Many studies have demonstrated the existence of biomarkers including genes, proteins, and pathways that can regulate aging and age-related diseases^32,40^. In this study, we also found that in high IMF content animal models (mouse and pigs), the expression of aging marker genes (*AHCY*, *GPNMB*, *VIM*) was increased. Overall, our results indicate a close association between fat infiltration in skeletal muscle and the aging process.

High-throughput single-cell and subcellular resolution transcriptomics have revolutionised the understanding of human organs and are increasingly utilized in the field of muscle research. Skeletal muscle function is governed by the niche compartment, and the collective action of individual cells within this compartment determines the biological function of skeletal muscle. However, it should be noted that some cell types in skeletal muscle are multinucleated (myofibers) while others (SCs, immune cells, fibroblasts, and adipocytes) are mononuclear. Hence, it has been technically challenging to explore potential functional distinctions and transcriptional heterogeneity in skeletal muscle. SnRNA-seq is a new high-resolution transcriptome method that is able to assay both mononuclear and multinucleated cells together^41^. Profiling the transcriptomes of mature muscle cells such as myotubes or myofibers can be performed at the whole cell or nucleus level by snRNA-seq. Recently, transcriptional heterogeneity in mammalian skeletal myofibers was identified by snRNA-seq^4,5^. Many cell types including SCs, FAPs, endothelial cells, tenocytes, smooth muscle cells, pericytes, immune cells, B/T cells, and adipocytes were identified in murine skeletal muscle. In the present study, we found that in aged human muscle, the niche compartment is composed of six different clusters, including myofibers, FAPs/fibroblasts, ECs, immune cells, MuSCs, and pericytes. Additionally, we also identified two specific clusters in the muscles of pigs: adipocytes and myeloid-derived cells. Previous studies also found that apart from slow myonuclei (type I myonuclei) and fast myonuclei (type IIa, IIx, and IIb myonuclei), myonuclei that clustered outside of fiber type included myotendinous junction (MTJ) and neuromuscular junction (NMJ) in adult mouse skeletal muscle^4,41^. However, we found MTJ is a conserved cluster only in pigs not in humans. NMJ is a small cluster of myonuclei responsible for the formation and maintenance of the synaptic apparatus^42^. In MTJ, there are many cell adhesion and cytoskeletal proteins and genes such as *Col22a1* and *Ankrd1*^43,44^. In aged humans, we found the NMJ marker gene *ABLIM2* was not specific which is different from high IMF content pigs. Although the violin plot and dot plot indicated the expression of MYH1 and MYH2 was also not specific in myofiber nuclei, we discovered *MYH2* and *MYH1* were significantly expressed in type IIa myonuclei and type IIx myonuclei in aged human muscles. Importantly, Petrany *et al*. found there were adipocyte clusters in the 24-month tibialis anterior of mouse^5^. Jing *et al*. also identified adipocyte clusters in the aged muscle of monkeys through snRNA-seq^28^. Similarly, our previous study also identified adipocyte clusters in pig muscles^31^. However, in this study, we did not detect adipocyte clusters in aged human muscles. This discrepancy may be attributed to individual and species differences. These results suggest that the skeletal muscle has great heterogeneity and many cell types can be found in aged skeletal muscle but there is still a large difference across species.

Animal models are critical in medical research on human disease and nutrition. Pigs have recently emerged as a biomedical model for multiple complex human diseases due to their closer anatomical and physiological characteristics to humans than small rodents such as mice^45^. Hence, pigs could serve as an alternative animal model to study the association between fat infiltration in skeletal muscle and aging. Our study compared the core gene program of myosteatosis in the aging across human and pigs. We found *MYBPC1*, *TRDN*, *ACTN2*, and *NRAP* are conserved genes in myofibers and strongly associatied with pericytes and MuSCs. These consensus signatures of humans and pigs may thus help increase reproducibility and reliability between future studies involving snRNA-seq in the muscle field.

The cellular origin of fat infiltration is manifold and recent studies have discovered several cell types leading to the formation of ectopic IMF^24,26^. IMF is mainly distributed in the endomysium, perimysium and epimysium of skeletal muscle, which are composed of phospholipids and triglycerides. For myogenic cells, MSCs are the main source of IMF and platelet-derived growth factor receptor α (PDGFRα)^+^ mesenchymal progenitors, which are distinct from SCs, have been considered contributing to ectopic fat cell formation^35^. We previously summarized that SCs have the potential capacity of transdifferentiating into adipocytes in skeletal muscle and this process is regulated by several factors including genes, nutrients, aging, regeneration stage and oxidative stress^25^. Besides, the existence of BAT derived from Myf5^+^ MSCs in skeletal muscle, and Pax7-Cre^ER^ linage tracing studies have demonstrated that PRDM16 can drive the formation of brown fat precursor-type cells from myoblasts or white fat precursors^46,47^. For non-myogenic cells, Uezumi *et al*.^35,48^ verified a subpopulation of non-myogenic MSCs constitutes the main source of intramuscular adipocytes during muscle regeneration in mice and humans, which is different from muscle SCs. This subpopulation of progenitor cells is PDGFRα^+^ or CD140α^+^ and has the biological potential of differentiating into lipid-rich adipocytes and expressing collagen I fibroblasts, which are defined as FAPs^35^. In our previous study, we found FAPs could differentiate into adipocytes in 2D and 3D cultured conditions^31^. In this study, we discovered the existence of FAPs in the muscles of aged humans and high IMF content pigs. These findings indicate FAPs are the main source of fat infiltration in skeletal muscle in mammalians. However, we found adipocyte population are not separate from the FAPs population in high IMF content pig muscles. Our previous study has found FAPs could directionally differentiate into PDE4D^+^/PDE7B^+^ adipocytes which is a subcluster in adipocytes cluster^31^. Interestingly, we also observed PDE4D^+^/PDE7B^+^ subclusters in aged human muscles and trajectory analysis showed PDGFRA^+^ FAPs could also differentiate into PDE4D^+^/PDE7B^+^ subclusters. Hence, that may be the reason why these cell populations cannot separate in high IMF content pig muscles. Based on these results, we speculate PDE4D^+^/PDE7B^+^ subclusters may be the preadipocytes which between FAPs and mature adipocytes, and play a vital role in IMF deposition. Besides, fibroblasts, pericytes and mesoangioblasts, SPs (also called myoendothelial cells), and PW1-expressing cells (PICs) also form IMF in the injured and inflamed skeletal muscle^24^. Specifically, our previous study revealed that IMF was mainly derived from FAPs and partly from CD68^+^ mononucleosus mononuclear myeloid cells in muscle^26^. In our study, we found ECs, fibroblasts, and pericytes are conserved clusters and SPs, myeloid-derived cells are specific clusters in aged human muscles and high IMF content pig muscles and these cell types interact with each other. Taken together, numerous kinds of cell types exist in human and pig muscles, and may have the potential capacity to contribute to fat infiltration in skeletal muscle tissues, but the sources of IMF deposition have species-specific characteristics.

In summary, this study revealed an association between fat infiltration in skeletal muscle and aging across species by performing an integrated analysis of bulk RNA-seq datasets. We also compared the myonuclear heterogeneity, different cell type populations, core genes program, and the transcriptomic differences of myosteatosis with human cell types with the help of data from the snRNA-seq of muscles in different species. The approach allowed us to identify core genes program and revealed conserved and differential genes between different species along fat infiltration in skeletal muscle. Our analysis reveals the potential cell biological rules of myosteatosis in aging across species, which may provide new targets for treating muscle-related diseases. Besides, the integration of resources on fat infiltration in skeletal muscle across species may promote the use of pigs as model organisms for human biological and biomedical studies.

## Methods

### Data acquisition

SnRNA-seq and RNA seq datasets generated from aged human muscle samples were downloaded from the Gene Expression Omnibus database (GEO Accession ID: GSE167186^49^, GSE129643^50^ and GSE136344^51^; https://www.ncbi.nlm.nih.gov/geo/) as count matrices^27,32,39^. The mass spectrometry proteomics datasets generated from aged human muscle samples were downloaded from the ProteomeXchange Consortium (ID: PXD011967)^32,52^. The collection consists of 4 snRNA-seq samples (sample ID: GSM5098740, GSM5098742, GSM5098750, GSM5098753). SnRNA-seq and RNA seq datasets generated from muscle of high IMF content pig samples were downloaded from the Genome Sequence Archive (Genomics, Proteomics & Bioinformatics 2021) in National Genomics Data Center (Nucleic Acids Res 2022), China National Center for Bioinformation/Beijing Institute of Genomics, Chinese Academy of Sciences (GSA: CRA011059 and CRA011069; https://ngdc.cncb.ac.cn/gsa)^31^. The collection consists of 2 snRNA-seq samples (sample ID: CRX704329, CRX704330). Sample information was carefully and manually checked by confirming in the original paper and details were shown in (Supplementary Fig. 1a and Table 1).

### RNA-seq analysis

We performed the re-analysis of the public RNA-seq datasets generated from aged human muscles, GLY-injected mouse muscles, and muscles of high IMF content pig samples^26,31,32,39^. FastQC (version 0.11.2) was used to evaluate the quality of the sequenced data. Raw reads were filtered by Trimmomatic (version 0.36) and clean reads were mapped to the reference genome used by HISAT2 (version2.0). Gene expression values of the transcripts were computed by StringTie (version 1.3.3b). Using the TPM to eliminate the effect of gene lengths and sequencing discrepancies to enable direct comparison of gene expression between samples. Comparisons were made by unpaired two-tailed Student’s t tests. Differences were considered significant at *P* < 0.05.

### Bioinformatics analysis

The SnRNA-seq results were demultiplexed and converted to FASTQ format using Illumina bcl2fastq software (version 2.20). Sample demultiplexing, barcode processing and single-cell 3′ gene counting were performed using the Cell Ranger pipeline (https://support.10xgenomics.com/single-cell-gene-expression/software/

overview/welcome, version 6.1.2). A total of 75434 single nuclei captured from 4 aged human and a total of 34443 single nuclei captured from 2 HLW pigs were processed using 10X Genomics Chromium Single Cell 3′ Solution. The Cell Ranger output was loaded into Seurat (version 4.1.0) for dimensional reduction, clustering, and analysis of snRNA-seq data. Overall, 47273 cells passed the quality control threshold: the number of genes expressed per cell > 500 and the percent of mitochondrial DNA-derived gene expression < 25%. The DoubletFinder package (version 2.0.3) was used to remove doublets. We also performed batch correction using Harmony for data integration between samples.

To visualize the data, we further reduced the dimensionality using Seurat and used UMAP to project the cells into 2D space. The steps include the following: 1. The LogNormalize method of the “Normalization” function of Seurat software was used to calculate the expression value of genes. 2. Principal component analysis (PCA) was performed using the normalized expression value. Among the PCs, the top 10 PCs were used to perform clustering and UMAP analysis. 3. To find clusters, the weighted shared nearest neighbour (SNN) graph-based clustering method was selected. Marker genes for each cluster were identified with the Wilcoxon rank-sum test with default parameters via the FindAllMarkers function in Seurat. This selects marker genes that are expressed in more than 10% of the cells in a cluster, *P* value ≤ 0.01, and average log2(fold change) ≥ 0.26. SnRNA data analysis was performed using the OmicStudio tools created by LC-BIO Co., Ltd (HangZhou, China) at https://www.omicstudio.cn/cell.

### Pseudotime analysis

To model differentiation trajectories, we performed trajectory analysis using Monocle 2 (http://cole-trapnell-lab.github.io/monocle-release/docs, version 2.22.0) as previously published^53^. Cell lineage trajectories of FAPs were inferred and characterized at the single-nucleus level. A UMI counts matrix was used as the input. The “newCellDataSet” function was used to create a CellDataSet object with parameter “expressionFamily = negbinomial.size()” following the Monocle2 tutorial. Based on significant differentially expressed genes (DEGs), the ‘Tobit’ expression family and ‘DDRTree’ reduction method were used with the default parameters. The cell lineage trajectory based on cell cluster and pseudotime was then inferred with the default parameters of Monocle2 after dimensionality reduction and cell ordering, then visualized with the “plot_cell_trajectory” function. Following cell trajectory, DEGs along pseudotime (named “pseudotime-dependent genes”) were found using the “differentialGeneTest” (fullModelFormulaStr = ~Pseudotime) function. Then “plot_genes_in_pseudotime” and “plot_pseudotime_heatmap” functions were used to visualize dynamic changes of pseudotime-dependent gene expression along pseudotime, and significant pseudotime-dependent genes were also visualized with a heatmap. Similarly, the BEAM test of Monocle 2 was applied to identify branch-dependent gene expression dynamically expressed along each branch, and significant branch-dependent genes were visualized with a heatmap.

### RNA velocity analysis

RNA velocity analysis was performed according to the previous study^54^. The generated bam files by Cell Ranger were sorted by SAMTools. The sorted bam files were then used to run the ‘run10x’ command from Velocyto to generate a loom file^55^. RNA velocity analysis was independently performed in FAPs.

### Cell‒cell communication analysis

For cell communication analysis, we used CellPhoneDB (https://www.cellphonedb.org, version 3.1.0) to further speculate on the underlying cellular interaction mechanisms. For each gene in a cell population, the expressed percentage and the average gene expression are calculated. Only when the proportion of cells which expressing receptor and ligand exceeds 10% will the ligand-receptor pair be included in the analysis. For the aggregates, the expressed value of the subunit with the lower expression average was selected to represent the expression of the receptor for subsequent statistical analysis. Pairwise comparisons were made for all cell types in the screened data set. First, all cells are randomly arranged to form a new cell population (the default number of random arrangements is 1000 times), the average expression level of ligands in the cell population after random arrangement and the average expression level of receptors in the cell types interacting with them are calculated, and then an average mean is obtained. This process is repeated many times and then gets a zero distribution of mean. After the zero distribution is generated, the actual mean value of the ligand-receptor pair between the two cell types is calculated according to the proto-cell population, and the possible significance *P*-value of the receptor-ligand pair in the two cell types is estimated based on the proportion of the calculated mean equal to or higher than the actual mean. Finally, highly specific interactions between cell types were sequenced based on the number of significant ligand-receptor pairs enriched in both cell types in order to manually screen for biologically relevant interactions.

### Pathway enrichment analysis

Gene Ontology (GO) and Kyoto Encyclopedia of Genes and Genomes (KEGG) enrichment analyses were used to identify which DEGs were significantly enriched in GO terms or metabolic pathways. GO terms and KEGG pathway analyses using the hypergeometric test were performed to identify significantly enriched metabolic pathways or signal transduction pathways enriched in DEGs. GO terms and KEGG pathways with false discovery rates *P* < 0.05 were considered significantly different.

### Statistical analysis

GraphPad (Prism 8.3.0) and R software (version 4.3.1) were used for data analyses and data visualization. Comparisons were made by unpaired two-tailed Student’s t tests. Differences were considered significant at *P* < 0.05.

### Supplementary information

Supplementary Figures

Supplementary Table 1

## Data Availability

All sequencing data reported in this paper have been deposited in the Gene Expression Omnibus database (GEO Accession ID: GSE167186, GSE129643 and GSE136344; https://www.ncbi.nlm.nih.gov/geo/) and the Genome Sequence Archive (Genomics, Proteomics & Bioinformatics 2021) in National Genomics Data Center (Nucleic Acids Res 2022), China National Center for Bioinformation/Beijing Institute of Genomics, Chinese Academy of Sciences (GSA: CRA011059 and CRA011069) that are publicly accessible at https://ngdc.cncb.ac.cn/gsa. The single-cell and single-nucleus datasets generated in the study have been deposited on Figshare^56^. The Figshare repository also contains supplementary Table 1 and supplementary Figs. 1, 2^57,58^.

## Code availability

The software required for data processing and analysis and image generation in this study are accessible, the software versions as follows:

1. Cell Ranger (version 6.1.2, https://support.10xgenomics.com/single-cell-gene-expression/software/overview/welcome).

2. OmicStudio (https://www.omicstudio.cn/cell).

3. Monocle 2 (version 2.22.0, http://cole-trapnell-lab.github.io/monocle-release/docs).

4. CellPhoneDB (version 3.1.0, https://www.cellphonedb.org).

## References

[1] Bentzinger CF, Wang YX, Rudnicki MA (2012). Building muscle: molecular regulation of myogenesis. Cold Spring Harb. Perspect. Biol..

[2] Zammit PS (2017). Function of the myogenic regulatory factors Myf5, MyoD, Myogenin and MRF4 in skeletal muscle, satellite cells and regenerative myogenesis. Semin. Cell Dev. Biol..

[3] Giordani L (2019). High-Dimensional Single-Cell Cartography Reveals Novel Skeletal Muscle-Resident Cell Populations. Mol. Cell.

[4] Dos Santos M (2020). Single-nucleus RNA-seq and FISH identify coordinated transcriptional activity in mammalian myofibers. Nat Commun.

[5] Petrany MJ (2020). Single-nucleus RNA-seq identifies transcriptional heterogeneity in multinucleated skeletal myofibers. Nat Commun.

[6] Miljkovic I, Zmuda JM (2010). Epidemiology of myosteatosis. Curr. Opin. Clin. Nutr. Metab. Care.

[7] Ahn H (2021). Updated systematic review and meta-analysis on diagnostic issues and the prognostic impact of myosteatosis: A new paradigm beyond sarcopenia. Ageing Research Reviews.

[8] Song MY (2004). Sarcopenia and increased adipose tissue infiltration of muscle in elderly African American women. Am. J. Clin. Nutr..

[9] Li CW (2022). Pathogenesis of sarcopenia and the relationship with fat mass: descriptive review. J Cachexia Sarcopenia Muscle.

[10] Biferali B (2021). Prdm16-mediated H3K9 methylation controls fibro-adipogenic progenitors identity during skeletal muscle repair. Sci Adv.

[11] Wosczyna MN (2021). Targeting microRNA-mediated gene repression limits adipogenic conversion of skeletal muscle mesenchymal stromal cells. Cell Stem Cell.

[12] Tieland M, Trouwborst I, Clark BC (2018). Skeletal muscle performance and ageing. J Cachexia Sarcopenia Muscle.

[13] Jiang Z, Marriott K, Maly MR (2019). Impact of Inter- and Intramuscular Fat on Muscle Architecture and Capacity. Crit. Rev. Biomed. Eng..

[14] Biltz NK (2020). Infiltration of intramuscular adipose tissue impairs skeletal muscle contraction. J. Physiol..

[15] Miljkovic I (2012). Skeletal Muscle Fat Infiltration Accelerates with Aging in African Ancestry Men. Gerontologist.

[16] Laurentius T (2019). Long-Chain Fatty Acids and Inflammatory Markers Coaccumulate in the Skeletal Muscle of Sarcopenic Old Rats. Dis. Markers.

[17] Bosch L, Tor M, Reixach J, Estany J (2012). Age-related changes in intramuscular and subcutaneous fat content and fatty acid composition in growing pigs using longitudinal data. Meat Sci.

[18] Uezumi A, Fukada S, Yamada H, Takeda S, Tsuchida K (2010). Mesenchymal progenitors distinct from muscle satellite cells contribute to ectopic fat cell formation in skeletal muscle. Differentiation.

[19] Pagano AF (2018). Short-term disuse promotes fatty acid infiltration into skeletal muscle. J Cachexia Sarcopenia Muscle.

[20] Uezumi, A. *et al*. Identification and characterization of PDGFR alpha(+) mesenchymal progenitors in human skeletal muscle. *Cell Death Dis*. **5**, 10.1038/cddis.2014.161 (2014). 10.1038/cddis.2014.161 PMC4001314 24743741

[21] Joe AW (2010). Muscle injury activates resident fibro/adipogenic progenitors that facilitate myogenesis. Nat. Cell Biol..

[22] Uezumi A, Fukada S, Yamamoto N, Takeda S, Tsuchida K (2010). Mesenchymal progenitors distinct from satellite cells contribute to ectopic fat cell formation in skeletal muscle. Nat. Cell Biol..

[23] Fitzgerald G (2023). MME(+) fibro-adipogenic progenitors are the dominant adipogenic population during fatty infiltration in human skeletal muscle. Commun Biol.

[24] Sciorati C, Clementi E, Manfredi AA, Rovere-Querini P (2015). Fat deposition and accumulation in the damaged and inflamed skeletal muscle: cellular and molecular players. Cell. Mol. Life Sci..

[25] Wang, L. Y. & Shan, T. Z. Factors inducing transdifferentiation of myoblasts into adipocytes. *J. Cell. Physiol*. 10.1002/jcp.30074 (2020).

[26] Xu Z (2020). Single‐cell RNA sequencing and lipidomics reveal cell and lipid dynamics of fat infiltration in skeletal muscle. Journal of Cachexia, Sarcopenia and Muscle.

[27] Perez K (2022). Single nuclei profiling identifies cell specific markers of skeletal muscle aging, frailty, and senescence. Aging (Albany N. Y.).

[28] Jing Y (2022). Protein Cell.

[29] Groenen MAM (2012). Analyses of pig genomes provide insight into porcine demography and evolution. Nature.

[30] Perleberg C, Kind A, Schnieke A (2018). Genetically engineered pigs as models for human disease. Dis. Model. Mech..

[31] Wang L (2023). Single-nucleus and bulk RNA sequencing reveal cellular and transcriptional mechanisms underlying lipid dynamics in high marbled pork. NPJ Sci Food.

[32] Ubaida-Mohien, C. *et al*. Discovery proteomics in aging human skeletal muscle finds change in spliceosome, immunity, proteostasis and mitochondria. *Elife* **8**, 10.7554/eLife.49874 (2019). 10.7554/eLife.49874 PMC6810669 31642809

[33] Cardoso AL (2018). Towards frailty biomarkers: Candidates from genes and pathways regulated in aging and age-related diseases. Ageing Res Rev.

[34] Wang LY, Valencak GT, Shan TZ (2024). Fat infiltration in skeletal muscle: Influential triggers and regulatory mechanism. iScience.

[35] Uezumi A, Fukada S, Yamamoto N, Takeda S, Tsuchida K (2010). Mesenchymal progenitors distinct from satellite cells contribute to ectopic fat cell formation in skeletal muscle. Nat. Cell Biol..

[36] Bourin P (2013). Stromal cells from the adipose tissue-derived stromal vascular fraction and culture expanded adipose tissue-derived stromal/stem cells: a joint statement of the International Federation for Adipose Therapeutics and Science (IFATS) and the International Society for Cellular Therapy (ISCT). Cytotherapy.

[37] Kim M (2020). Single-nucleus transcriptomics reveals functional compartmentalization in syncytial skeletal muscle cells. Nat Commun.

[38] Ubaida-Mohien C (2019). Discovery proteomics in aging human skeletal muscle finds change in spliceosome, immunity, proteostasis and mitochondria. Elife.

[39] Gueugneau, M. *et al*. Muscle Proteomic and Transcriptomic Profiling of Healthy Aging and Metabolic Syndrome in Men. *Int. J. Mol. Sci*. **22**, 10.3390/ijms22084205 (2021). 10.3390/ijms22084205 PMC8074053 33921590

[40] Kapitansky O, Gozes I (2019). ADNP differentially interact with genes/proteins in correlation with aging: a novel marker for muscle aging. Geroscience.

[41] Chemello F (2020). Degenerative and regenerative pathways underlying Duchenne muscular dystrophy revealed by single-nucleus RNA sequencing. Proc. Natl. Acad. Sci. USA.

[42] Li L, Xiong WC, Mei L (2018). Neuromuscular Junction Formation, Aging, and Disorders. Annu. Rev. Physiol..

[43] Koch M (2004). A novel marker of tissue junctions, collagen XXII. J. Biol. Chem..

[44] Charvet B (2013). Knockdown of col22a1 gene in zebrafish induces a muscular dystrophy by disruption of the myotendinous junction. Development.

[45] Jin L (2021). A pig BodyMap transcriptome reveals diverse tissue physiologies and evolutionary dynamics of transcription. Nat Commun.

[46] Yin H (2013). MicroRNA-133 controls brown adipose determination in skeletal muscle satellite cells by targeting Prdm16. Cell Metab..

[47] Seale P (2008). PRDM16 controls a brown fat/skeletal muscle switch. Nature.

[48] Uezumi A (2014). Identification and characterization of PDGFRalpha+ mesenchymal progenitors in human skeletal muscle. Cell Death Dis..

[49] Perez K (2022). GEO..

[50] Ubaida-Mohien C (2020). GEO..

[51] Gueugneau M (2021). GEO..

[52] Ubaida-Mohien C (2020). GEO..

[53] Huang XZ (2023). Single-cell sequencing of ascites fluid illustrates heterogeneity and therapy-induced evolution during gastric cancer peritoneal metastasis. Nat Commun.

[54] La Manno G (2018). RNA velocity of single cells. Nature.

[55] Bergen V, Lange M, Peidli S, Wolf FA, Theis FJ (2020). Generalizing RNA velocity to transient cell states through dynamical modeling. Nat. Biotechnol..

[56] Wang LY (2024). Figshare..

[57] Wang LY (2024). Figshare..

[58] Wang LY (2024). Figshare..

