# Supplementary material for: Integrative cross-species analysis reveals conserved and unique signatures in fatty skeletal muscles
Source: Sci Data. 2024 Mar 12;11:290. doi: 10.1038/s41597-024-03114-5 (PMC10933306; doi:10.1038/s41597-024-03114-5)
Supplement: Supplementary file 1 — Supplementary Figures [file 41597_2024_3114_MOESM1_ESM.pdf]

Supplementary Information for

Integrative cross-species analysis reveals conserved and unique signatures in fatty skeletal muscles

Liyi Wang, Yanbing Zhou, Yizhen Wang, Tizhong Shan

***Corresponding authors: Tizhong Shan (tzshan@zju.edu.cn)**

This PDF file includes:

Supplementary Figure 1. Individual characteristics of the samples included in the analysis......2

Supplementary Figure 2. The expression of selected signature genes for each subcluster in myofibers nuclei. 3

Supplementary Figure 3. Pseudotime trajectory analysis of FAPs nuclei by RNA velocity. 4

a

Sample name	GEO accession number		Gender	Age	Tissue
	Series	Sample ID			
**oldHM4**	GSE167186	GSM5098740	Male	79	Vastus lateralis
**oldHM6**	GSE167186	GSM5098742	Male	82	Vastus lateralis
**oldHM15**	GSE167186	GSM5098750	Male	79	Vastus lateralis
**oldHM23**	GSE167186	GSM5098753	Male	90	Vastus lateralis
**HLW1**	CRA011059	CRX704329	Male	adult	Longissimus dorsi muscle
**HLW2**	CRA011059	CRX704330	Male	adult	Longissimus dorsi muscle

b

Sample	Estimated Number of Cells	Fraction Reads in Cells	Mean Reads per Cell	Median Genes per Cell	Median UMI Counts Per Cells
**oldHM4**	38297	78.0%	11448	300	483
**oldHM6**	9549	61.7%	50018	517	823
**oldHM15**	3951	41.9%	95596	844	1637
**oldHM23**	23637	74.7%	28502	841	1539
**HLW1**	13836	77.6%	25070	1162	2066
**HLW2**	20607	77.0%	16430	857	1276

Supplementary Figure 1. Individual characteristics of the samples included in the analysis. (a) Sample information in aged human and high IMF content pig muscles. **(b)** The results obtained from Cell Ranger analyses.

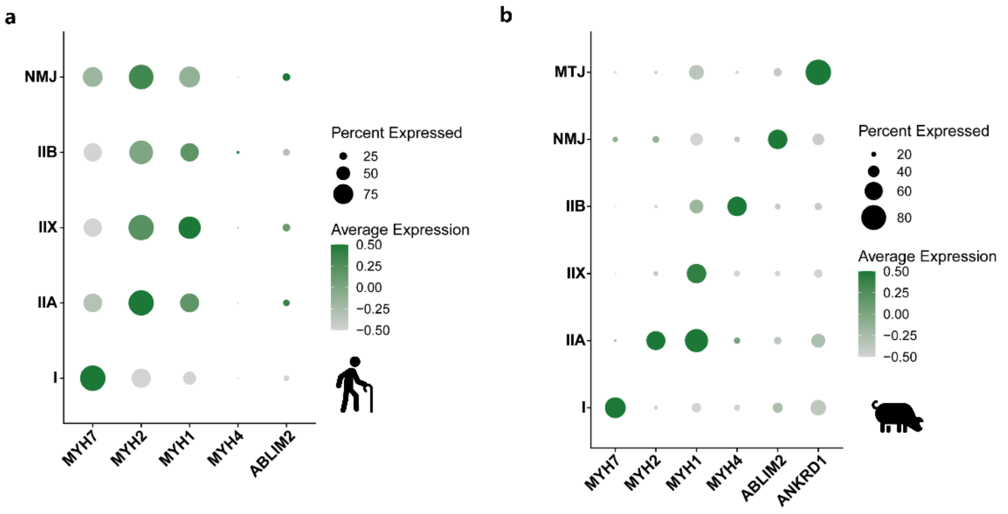

Supplementary Figure 2. The expression of selected signature genes for each subcluster in myofibers nuclei. (a) Dotplot showing the expression of selected marker genes for each subcluster of myofibers nuclei in aged human muscles. **(b)** Dotplot showing the expression of selected marker genes for each subcluster of myofibers nuclei in high IMF content pig muscles.

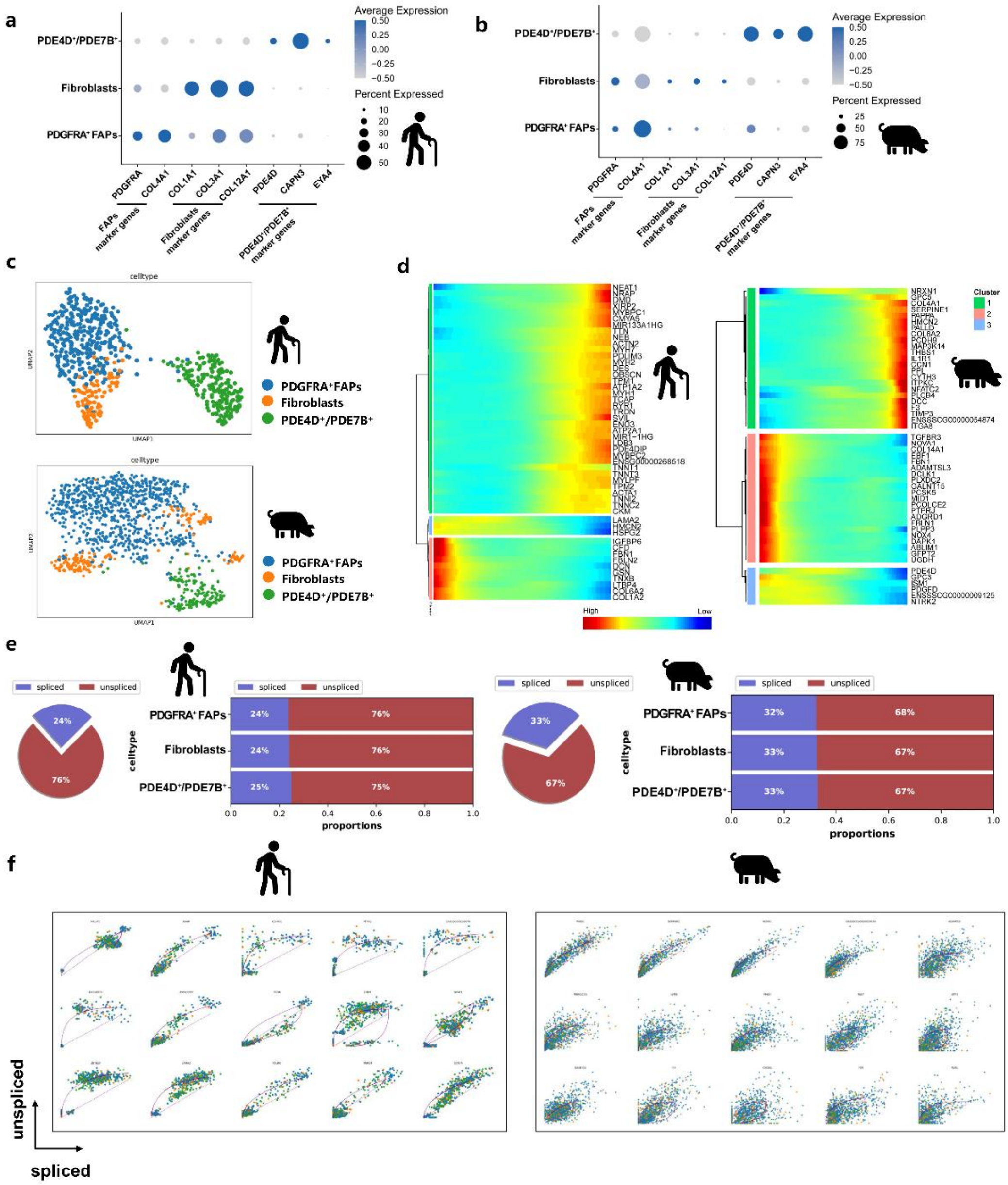

Supplementary Figure 3. Pseudotime trajectory analysis of FAPs nuclei by RNA velocity. **(a)** Dotplot showing the expression of FAPs marker genes, Fibroblasts marker genes, and PDE4D^{+}/PDE7B^{+} marker genes in aged human muscles. **(b)** Dotplot showing the expression of FAPs marker genes, Fibroblasts marker genes, and PDE4D^{+}/PDE7B^{+} marker genes in high IMF content pig muscles. **(c)** Distribution of the three subtypes on the UMAP. **(d)** Pseudotemporal heatmap showing gene expression dynamics for significant marker genes. Genes (rows) were clustered into three modules, and cells (columns) were ordered according to pseudotime in different species. The horizontal coordinate represents pseudotime, increasing from left to right, and the vertical coordinate represents pseudotime-related differential genes. The color represents the gene expression, red to blue which means the higher gene expression to the lower gene expression. **(e)** Pie chart showing the proportion of FAPs that are spliced versus un-spliced. Bar plot showing the proportion of FAPs subtypes that are spliced versus un-spliced in different species. **(f)** Transcriptional dynamics of top 15 marker genes on the UMAPs based on RNA velocity analysis calculated differences by t-test.
